# Operative Dentistry

**Published:** 1869-06

**Authors:** J. G. Willis


### OPERATIVE DENTISTRY.

BY J. G. WILLIS, M. D.

Operative Dentistry is conservative in its nature; its mission is to save, not to destroy; to retain, and not to restore. Therefore every new discovery or improvement made in the treatment of the natural teeth when diseased, which enables the Dentist more certainly to save them, is an additional laurel in the crown of Operative Dentistry. Successful Operative Dentistry is directly opposed to Mechanical Dentistry, which has been the fruitful cause of the destruction of myriads of valuable teeth. After viewing the subject from every stand-point, I am forced to the conclusion that if Mechanical Dentistry was blotted out of existence to-day, future generations would be largely benefited. The facility and cheapness with which artificial teeth can be supplied, induces hundreds of persons—yes, thousands, to neglect their natural teeth, consoling themselves with the reflection that when lost they can procure a new set. The amount of plate-work done by the profession in the aggregate in this country is a burning disgrace to the *science* of Dentistry.

The inventive genius of multitudes of men is engaged in the production of new and hitherto unused materials for bases for artificial dentures, proving the increasing demand for them, and demonstrating the immense sacrifice of the natural organs which is being made hourly all over this land, to make room for them. This should not be so. Every year, as scientific light and information are disseminated, the demand for artificial teeth should grow less and less, until they should be demanded ultimately by those only, whose need of them has been caused by accident.

Every Dentist has a sacred duty to perform, and *he* neglects his *first* duty to his patients, who does not persistently urge upon them the utmost importance of using all the care and means in their power to preserve their natural teeth. A successful surgeon prides himself upon the infrequency of wooden legs and arms among his patrons, and he advises amputation, only as a dernier resort, after every possible means have been used to save the member, and life is threatened by failure of them all. False teeth then, may be regarded as bearing the same relation to conservative Dentistry, that false legs and arms do to conservative Surgery. Sometimes surgeons are called upon in Courts of Justice to show and demonstrate, that from the condition of the member, amputation was imperatively demanded and unavoidable. I very much fear, that if an inspection of many of the teeth, removed to make room for artificial substitutes could be made, they would be found to be amenable to treatment by which they could have been retained, and proved of lasting service to their possessors.

If false teeth were more expensive, and in other respects more difficult to procure, people would be more likely to take better care of their natural teeth, and endeavor to retain them. Cheap teeth have got a great deal to answer for, and they who insert the most and pride themselves on the fact, need to remember, that their success in the *art* is a direct imputation upon their *science.* If every Dentist in the land would persistently refuse to remove a sound tooth, or one that could be saved by treatment and plugging, this now great evil would be reduced to its smallest possible proportions. Then indeed would the science of Dentistry have the greatest claim upon the people for their support, and its beneficent results would be *more* frequently seen in the mouths of the public at large than at present.

